# Diagnosis, management, and outcome of cardiac sarcoidosis and giant cell myocarditis: a Swedish single center experience

**DOI:** 10.1186/s12872-022-02639-0

**Published:** 2022-04-26

**Authors:** Emanuele Bobbio, Clara Hjalmarsson, Marie Björkenstam, Christian L. Polte, Anders Oldfors, Ulf Lindström, Pia Dahlberg, Sven-Erik Bartfay, Piotr Szamlewski, Amar Taha, Egidija Sakiniene, Kristjan Karason, Niklas Bergh, Entela Bollano

**Affiliations:** 1 Departments of Cardiology, Sahlgrenska University Hospital, Gothenburg, Sweden; 2 Departments of Clinical Physiology and Radiology, Sahlgrenska University Hospital, Gothenburg, Sweden; 3 Departments of Clinical Pathology, Sahlgrenska University Hospital, Gothenburg, Sweden; 4 Departments of Rheumatology, Sahlgrenska University Hospital, Gothenburg, Sweden; 5 Transplant Institute, Sahlgrenska University Hospital, Gothenburg, Sweden; 6 Institute of Medicine at Sahlgrenska Academy, University of Gothenburg, Gothenburg, Sweden

**Keywords:** Cardiac sarcoidosis, Giant cell myocarditis, Inflammatory cardiomyopathy, Myocarditis, Endomyocardial biopsy, Heart failure

## Abstract

**Background:**

Cardiac sarcoidosis (CS) and giant cell myocarditis (GCM) are rare diseases that share some similarities, but also display different clinical and histopathological features. We aimed to compare the demographics, clinical presentation, and outcome of patients diagnosed with CS or GCM.

**Method:**

We compared the clinical data and outcome of all adult patients with CS (n = 71) or GCM (n = 21) diagnosed at our center between 1991 and 2020.

**Results:**

The median (interquartile range) follow-up time for patients with CS and GCM was 33.5 [6.5–60.9] and 2.98 [0.6–40.9] months, respectively. In the entire cohort, heart failure (HF) was the most common presenting manifestation (31%), followed by ventricular arrhythmias (25%). At presentation, a left ventricular ejection fraction of < 50% was found in 54% of the CS compared to 86% of the GCM patients (*P* = 0.014), while corresponding proportions for right ventricular dysfunction were 24% and 52% (*P* = 0.026), respectively. Advanced HF (NYHA ≥ IIIB) was less common in CS (31%) than in GCM (76%). CS patients displayed significantly lower circulating levels of natriuretic peptides (*P* < 0.001) and troponins (*P* = 0.014). Eighteen percent of patients with CS included in the survival analysis reached the composite endpoint of death or heart transplantation (HTx) compared to 68% of patients with GCM (*P* < 0.001).

**Conclusion:**

GCM has a more fulminant clinical course than CS with severe biventricular failure, higher levels of circulating biomarkers and an increased need for HTx. The histopathologic diagnosis remained key determinant even after adjustment for markers of cardiac dysfunction.

**Supplementary Information:**

The online version contains supplementary material available at 10.1186/s12872-022-02639-0.

## Introduction

Cardiac sarcoidosis (CS) and giant cell myocarditis (GCM) are rare and serious forms of inflammatory cardiomyopathies characterized by highly variable manifestations and prognosis [1–3]. Despite extensive research, the etiology and pathophysiology of these conditions remain largely elusive [1, 4]. Endomyocardial biopsy is the gold standard for diagnosing both CS and GCM. However, their differentiation is still challenging and susceptible of confusion due to several shared histopathological and clinical features [5]. Their initial clinical manifestations can include heart failure (HF), fatal or life-threatening ventricular tachyarrhythmias, and conduction abnormalities [6]. Hence, the presentation of GCM is more aggressive and portends a worse prognosis if not correctly diagnosed and treated [7]. Whether CS and GCM should be considered to be different disease entities or two manifestations of the same disorder is still a matter of debate [6]. The hypothesis that the disorders can be considered to be two expressions of a single disease continuum has persisted for decades [8].

Sarcoidosis is more common in Sweden than in other countries with an incidence of 11/100,000 and a prevalence of > 50/100,000 [9, 10]. Information concerning the incidence and prevalence of GCM is not available, likely due to rarity of the disease and a declining rate of autopsies.

The aim of this study was to analyze and compare the demographics, cardiac manifestations, imaging/laboratory examination findings, and long-term outcome of patients with CS and GCM diagnosed between 1991 and 2020 at a large academic cardiac center in Sweden.

## Methods

### Study population and data retrieving

All adult patients diagnosed with CS or GCM at the Department of Cardiology, Sahlgrenska University Hospital in Gothenburg, Sweden, between 1991 and 2020 were retrospectively identified from the local hospital discharge registry. Data were corroborated with information extracted from the national pacemaker/implantable cardioverter defibrillator (ICD)-registry and reports of the pathology analysis of performed myocardial biopsies. Hospital charts were scrutinized for data on patient demographics, onset symptoms, imaging findings, laboratory analyses, invasive procedures, and details of medical and device treatments. Records of follow-up visits were also reviewed in order to collect data on outcomes, including heart transplantation (HTx) and death. This study conforms to the principles outlined in the Declaration of Helsinki and was approved by the Ethic Review Authority (Approval No 2019-05401).

### Diagnostic criteria

According to current recommendations from the Heart Rhythm Society (HRS) and Japanese Circulation Society (JCS) histological diagnosis was made when positive myocardial biopsy was obtained or, when not available, documentation of extra-cardiac histology of sarcoidosis associated with both clinical manifestations of myocardial involvement and abnormalities consistent with CS in ^18^-F-fluorodeoxyglucose positron emission/computed tomography (^18^F-FDG PET/CT), cardiac magnetic resonance imaging (CMR) or echocardiography [11–13]. If a positive biopsy result was not obtained in other organs either, a “clinical” diagnosis of CS was enabled based on the clinical manifestations and imaging findings according to the JCS guidelines [13]. These “clinical” CS diagnoses were previously diagnosed as “probable” CS based on the recommendations of the HRS 2014 statement [11]. The presence of non-necrotizing epithelioid cell granulomas with isolated giant cells and the absence of both, considerable myocardial necrosis and abundant tissue eosinophilia, were required for the histological diagnosis of sarcoidosis [14, 15].

The diagnosis of GCM was confirmed by a histological examination of a myocardial biopsy in all patients and required the presence of a widespread inflammatory infiltrate with multinucleated giant cells in association with myocyte damage [16]. To minimize the risk of misdiagnosis due to an acknowledged histological overlap between CS and GCM [16], all myocardial tissue samples were re-evaluated by a highly-experienced cardiac pathologist (A.O.). The International Classification of Disease, 10th revision codes were used to define prevalent cardiac manifestations: I47.2 (ventricular tachycardia), I49.0 (ventricular fibrillation), I50 (heart failure), R00.1 (bradycardia), I44.1 or I44.2 (second- or third-degree atrioventricular block), I45.3 (trifascicular block), I46 (cardiac arrest), or others [R50.9 (fever); R53 (fatigue); and R42 (dizziness)].

### Diagnostic work-up

A 12-lead surface electrocardiography recorded at clinical presentation was interpreted by an experienced cardiologist. The following parameters were recorded: heart rhythm and rate, PQ interval, second- or third-degree atrioventricular block, complete right bundle branch block or left bundle branch block, pathological Q waves, or frequent premature ventricular complexes.

The echocardiographic examination (Vivid E9 or E95, GE Healthcare, Milwaukee, Wisconsin, USA) was performed at presentation and was re-analyzed offline by an experienced cardiologist blinded to the final diagnosis. The left ventricular (LV) systolic function was assessed using the LV ejection fraction and volumes by the biplane Simpsons method. In order to assess LV diastolic function four variables were evaluated: annular e’ velocity (range of normal: septal e’ < 7 cm/s, lateral e’ < 10 cm/s), average E/e’ ratio (range of normal: > 14), left atrial maximum volume index (range of normal: > 34 mL/m^2^), and peak tricuspid regurgitation (TR) velocity (range of normal: > 2.8 m/s). LV diastolic dysfunction was present if more than half of the available parameters fell outside these cutoff values [17]. Right ventricular (RV) dysfunction was diagnosed when at least one of the following measurements fell outside the recommended range of normal: fractional area change (FAC) < 35%; tricuspid annular plane systolic excursion (TAPSE) < 17 mm; and Doppler tissue imaging-derived systolic S′ velocity of the tricuspid annulus < 9.5 cm/s [18]. Pulmonary artery systolic pressure (PASP) was estimated using the TR jet velocity and estimated central venous pressure [19, 20].

CMR studies were performed using a 1.5-T magnetic resonance imaging scanner (Gyroscan Intera or Achieva; Philips Healthcare, Best, The Netherlands) with a cardiac-dedicated phased-array coil, under electrocardiogram gating, and breath-holding in line with standard recommendations [21]. LV volumes were obtained by manual tracing of the epicardial and endocardial contour in end-diastole in the short axis (SA) slices of the continuous SA stack, propagated through all phases using a semi-automated tracing algorithm. The ejection fraction was calculated as [(end-diastolic volume minus end-systolic volume)/end-diastolic volume] × 100%. Ten minutes after an intravenous injection of 0.2 mmol/kg gadolinium, inverse recovery sequences were obtained to assess for the presence of gadolinium-enhancing lesions (LGE). The optimal inversion recovery time was defined as previously described [21].

### Statistical analysis

Descriptive statistics were used to account for demographic and clinical characteristics. Data are presented as median (Interquartile Range), or numbers (percentages). Comparisons between baseline data of patients with CS and GCM were performed with Mann–Whitney *U* test for continuous variables and Fisher’s exact test for categorical variables. Baseline was set at the time of the first clinical manifestation/symptom considered to be consistent with the diagnosis of either CS or GCM. The main outcome was defined as a composite endpoint of death or HTx. Survival was analyzed by Kaplan–Meier curves and the two groups were compared with the log-rank test. To assess the impact of CS versus GCM on prognosis and identify patient characteristics predictive of outcome, both univariate and multivariate Cox regression analyses were conducted. Variables with *P* ≤ 0.005 in the univariate analysis were included in the multivariate Cox regression model. Three models were tested, to minimize the risk of multicollinearity; covariates with R-coefficients > 0.7 (according to Spearman’s correlation) were not input in the analyses. Six patients with CS and 2 GCM were excluded from survival analysis owing to lack of follow-up time since they were diagnosed at the time of transplantation, implantation of mechanical circulatory support (MCS), or autopsy. To examine trends statistically over time we employed Poisson regression. All statistical tests were two-tailed (alpha level 0.05) and *P*-values < 0.05 were considered statistically significant. Statistical analyses were performed with SPSS 25.0 statistical software packages (SPSS Inc. Chicago, IL).

## Results

### Patient characteristics and clinical presentation

Seventy-one patients with CS and 21 patients with GCM were included in the present study. The diagnosis CS was confirmed in a myocardial sample in thirty-three patients (46%), whereas 22 (32%) had extra-cardiac biopsies indicative of sarcoidosis and clinical manifestations consistent with the disease. Sixteen patients (22%), with clinical manifestations of CS, met diagnostic criteria based on abnormal ^18^F-FDG PET and CMR at the time of diagnosis. Extra-cardiac sarcoidosis was detected in 72% of the patients, most commonly in the lungs (49%), followed by engagement of lymph nodes (13%) and involvement of the skin (4%). In all patients without signs of extra-cardiac involvement (28%) CS was confirmed in a sample of the myocardium.

Clinical and demographic characteristics of the entire cohort are presented in Table 1. At baseline, the median (Interquartile Range, IQR) age for CS and GCM was 56 (49–60) and 53 (46–63) years, respectively (*P* = 0.442). Patients with CS had higher body mass index [27.2 (24.2–30.6) vs 23.3 (21.2–27.6), *P* = 0.004], and a lower prevalence of autoimmune diseases (1% vs 19%, *P* = 0.009) compared with GCM. Moreover, patients with CS displayed significantly lower circulating levels of natriuretic peptides (*P* < 0.001), troponins (*P* = 0.014), and serum creatinine (*P* < 0.001) (Table 1). Prevalent cardiac manifestations at presentation were similar among patients with CS and GCM. In the entire cohort, the most common presenting manifestation was heart failure (31%), followed by ventricular arrhythmias (25%), and high-grade atrioventricular block (21%) (Table 1).

**Table 1:** Clinical and demographic characteristics of the whole study cohort and participants with CS and GCM at presentation

	All patients (n = 92)	CS (n = 71)	GCM (n = 21)	*p*
Age (years)	55 (48–60)	56 (49–60)	53 (46–63)	0.442
Female Gender	31 (34)	20 (29)	11 (52)	0.064
BMI (kg/m^2^)	26.4 (23.1–30.5)	27.2 (24.2–30.6)	23.3 (21.2–27.6)	0.004
NYHA class ≥ III	38 (41)	22 (31)	16 (76)	< 0.001
*Comorbidities and laboratory findings*				
Hypertension	31 (33)	25 (35)	6 (29)	0.793
Diabetes mellitus	7 (8)	6 (8)	1 (5)	1
Previous CVD^#^	13 (14)	12 (17)	1 (5)	0.285
Thyroid disease	11 (12)	8 (11)	3 (14)	0.709
Autoimmune Disease	5 (5)	1 (1)	4 (19)	0.009
NT-proBNP (pg/mL)§	1060 (279–5850)	808 (222–2425)	8309 (3562–24,482)	< 0.001
Troponin T (ng/L)*	34 (12–142)	27.6 (8–55)	473 (128–1270)	0.014
Creatinine (mg/dL)	97 (82–130)	93 (76–115)	134 (107–181)	< 0.001
eGFR (ml/min/1.73 m^2^)	70 (49–80)	73 (56–82)	54 (30–75)	0.016
*Prevalent cardiac manifestations at presentation*				
Heart failure	29 (31)	22 (31)	7 (33)	1
Sustained VT or VF	23 (25)	15 (21)	8 (38)	0.152
High-grade AVB	19 (21)	17 (24)	2 (9)	0.223
Sudden cardiac arrest	6 (6)	5 (7)	1 (5)	1
Chest pain	8 (9)	5 (7)	3 (14)	0.377
Other symptoms or signs^¶^	6 (6)	6 (8)	0	0.330

Data are numbers (%) of cases; medians (interquartile range)

AVB, Atrio-ventricular block; BMI, Body mass index; CVD, Cardiovascular diseases; CS, Cardiac sarcoidosis; eGFR, Estimated Glomerular filtration rate by CKD-EPI equation; GCM, Giant cell myocarditis; NYHA, New York Heart Association; VT, Ventricular tachycardia; VF, Ventricular fibrillation

^#^Including: supraventricular arrhythmias, transient ischemic attack, aortic aneurism

^*^Data reported on 50 patients (54% of the entire cohort, 50% of the CS group, 71% of the GCM group)

^§^Data reported on 82 patients (90% of the entire cohort, 89% of the CS group, 90% of the GCM group)

^¶^Including: fever, fatigue, and dizziness

**Fig. 1:**
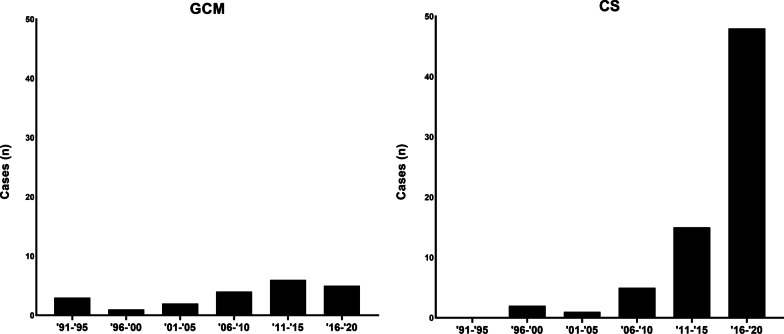
The number of new cases of CS and GCM diagnosed in the 5-year periods between 1991 and 2020

The number of newly diagnosed CS cases increased significantly over time (*P* < 0.001), whereas the incidence of GCM remained largely unchanged (Fig. 1).

### Imaging findings and diagnosis

Findings from echocardiography and CMR examinations at disease presentation are shown in Table 2. Patients with GCM displayed impaired LV and RV systolic function more often than those with CS, as well as LV diastolic dysfunction, but no differences were observed with respect to LV dimensions. Mild pulmonary hypertension was more common in patients with GCM. The heart rate was lower in CS patients than in those with GCM, but otherwise the ECG was similar between the two groups.

**Table 2:** Findings from electrocardiography, echocardiography, and cardiovascular magnetic resonance imaging examinations at presentation of the whole study cohort and participants with CS and GCM

	All patients (n = 92)	CS (n = 71)	GCM (n = 21)	*p*
*ECG*				
Atrial fibrillation	3 (30)	2 (3)	1 (5)	0.512
HR (bpm)	65 (54–78)	62 (50–77)	75 (63–93)	0.007
PQ (ms)	180 (160–212)	186 (151–212)	175 (163–224)	0.947
High-grade AVB	19 (21)	17 (24)	2 (9)	0.223
Right BBB	21 (23)	16 (23)	5 (24)	0.764
Left BBB	11 (12)	11 (15)	4 (19)	0.108
Q-wave	5 (5)	3 (4)	2 (10)	0.281
Frequent PVCs	17 (18)	11 (15)	6 (28)	0.176
*Echocardiography*				
LV EF (%)	40 (27–55)	45 (30–55)	30 (20–41)	0.002
LV ED diameter (mm)	56 (51–62)	56 (51–62)	54 (51–61)	0.360
LV ED volume (ml)	139 (107–181)	155 (108–209)	124 (105–147)	0.055
LV DD	36 (39.1)	22 (31)	14 (67)	0.010
RV dysfunction	28 (30.4)	17 (24)	11 (52)	0.026
sPAP (mmHg)	30 (25–40)	29 (23–40)	40 (30–45)	0.050
*MRI*				
	N = 58	N = 46	N = 12	
LV ED volume (ml)	171 (135–232)	162 (130–232)	193 (159–249)	0.075
LV ES volume (ml)	139 (85–181)	90 (76–203)	150 (105–157)	0.376
LV EF (%)	43 (32–57)	50 (35–60)	30 (22–42)	0.022
RV ED volume (ml)	162 (123–225)	158 (97–217)	188 (139–247)	0.741
RV ES volume (ml)	106 (77–200)	101 (58–227)	119 (92–186)	0.252
RV EF (%)	48 (26–60)	52 (39–62)	29 (24–45)	0.031
Delayed enhancement	54 (93)	43 (93)	11 (92)	1

Data are numbers (%) of cases; medians (interquartile range)

AVB, Atrio-ventricular block; BBB, Bundle branch block; HR, Heart rate; LV DD, Left ventricular diastolic dysfunction; LV ED, Left ventricular end-diastolic; LV EF, Left ventricular ejection fraction; LV ES, Left ventricular end-systolic; PVCs, Premature ventricular complexes; RV, Right ventricular; RV ED, Right ventricular end-diastolic; RV EF, Right ventricular ejection fraction; RV ES, Right ventricular end-systolic; sPAP, systolic pulmonary artery pressure

### Medical treatment

All patients, except those diagnosed by histological examination of the explanted heart after HTx, received disease-modifying immunosuppressive therapy. Patients with CS underwent treatment with steroids inducing an initial prednisone-equivalent dose varying from 30 to 60 mg daily tapering to < 10 mg, usually 2.5–5 mg daily after 6 months. In 52 of the 63 patients (83%), steroid use was uninterrupted until the end of follow-up, HTx, or death. Prednisone-equivalent dose was temporarily increased in 8 patients because of disease reactivation during follow-up. In addition to and in many cases to reduce steroids, methotrexate was used in 27 patients, infliximab in 4 patients, azathioprine in 3 patients, and adalimumab in 1 patient. All 19 patients with an ultimate diagnosis of GCM presenting alive were administered steroids, in combination with cyclosporine in 15 patients, mycophenolate mofetil in 5 patients, and azathioprine in 4 patients. An ICD was implanted in 46 patients with CS (65%), and in 11 patients with GCM (52%). A pacemaker due to atrioventricular conduction block was implanted in 14 patients with CS (20%) and in 5 patients with GCM (24%).

### Outcome

During a median follow-up time of 33.5 [6.5–60.9] months, 18 of the 71 patients (25%) with CS reached the composite endpoint of HTx or death. Six patients with CS, who were diagnosed at the time of transplantation, MCS implantation or autopsy were excluded from the survival analysis. Among the remaining 65 CS patients, 2 patients died and 11 patients received a heart transplant, of whom 5 (45%) were bridged to transplantation with a Left Ventricular Assist Device (LVAD).

The median follow-up time of GCM patients was 2.98 [0.6–40.9] months. The composite endpoint of HTx or death was reached in 15 patients (71%). Two cases were excluded from the survival analysis due to the diagnosis of GCM being made in the explanted heart at the time of HTx. Two GCM patients died and the remaining 11 patients underwent HTx; of these, 5 (45%) were bridged to transplant with an LVAD.

**Fig. 2:**
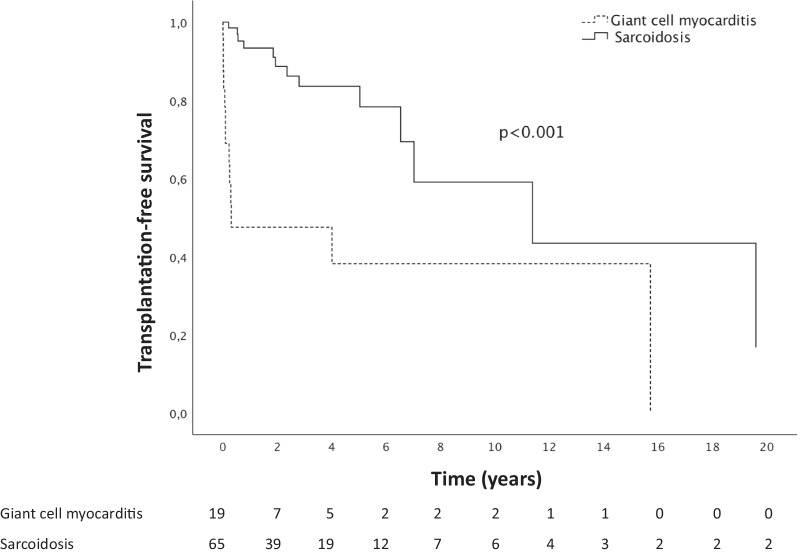
Kaplan–Meier curves for transplantation-free survival in patients with cardiac sarcoidosis and giant cell myocarditis

The Kaplan–Meier curves for event-free survival for the 2 groups are shown in Fig. 2. The graphs show a rapid divergence, with most events in GCM occurring between 0.5 and 4 years from disease onset. Clinical and demographic characteristics, as well as imaging findings of the patients included in the survival analysis are presented in Additional file 1: Table S1 and Additional file 2: Table S2.

During follow-up, sustained ventricular tachycardia was recorded in 13 CS and 2 GCM patients leading to implantation of an ICD. However, these arrhythmia-events were not included in the outcome analyses.

In the univariate analysis, male gender, GCM diagnosis, eGFR, natriuretic peptide levels, as well as NYHA class ≥ III, left ventricular EF, and right ventricular dysfunction were associated with worse outcome. However, due to significant correlation to all other covariates of interest, eGFR was not input in the Cox proportional regression. In the multivariate analyses, male gender and GCM diagnosis were independently associated with survival in two of the three models; NYHA class ≥ III as well as reduced left ventricular EF were found to independently predict poor outcome; in model 3, only 20 events were included, due to few missing NTproBNP-values (Table 3).

**Table 3:** Univariate and multivariate Cox proportional hazard regression evaluating the risk of endpoint (death or heart transplantation) in the entire cohort

	Univariate	Multivariate					
	*p*	Model 1 N patients = 72 N events = 23		Model 2 N patients = 71 N events = 23		Model 3 N patients = 65 N events = 20	
		HR [CI]	*P*	HR [CI]	*P*	HR [CI]	*P*
*Baseline characteristics*							
Age (years)	0.564						
Male gender	0.004	3.11 [1.20–8.05]	0.019	4.11 [1.65–10.23]	0.002	2.84 [0.98–8.26]	0.055
BMI (kg/m^2^)	0.151						
GCM diagnosis	< 0.001	3.61 [1.42–9.21]	0.007	3.47 [1.37–8.79]	0.009	2.69 [0.81–8.94]	0.106
NT-proBNP (pg/L)	< 0.001					1.00 [1.00–1.00]	0.259
eGFR (ml/min/1.73 m^2^)	0.008						
NYHA class ≥ III	< 0.001			3.38 [1.13–10.12]	0.03		
*Prevalent clinical cardiac manifestations*							
Heart failure	0.094						
Sustained VT or cardiac arrest	0.576						
High-grade AVB	0.06						
*Echocardiography findings*							
LV EF (%)	< 0.001	0.95 [0.91–0.99]	0.01				
RV dyfunction	0.005	1.29 [0.47–3.56]	0.627	1.67 [0.64–4.35]	0.293	2.39 [0.82–7.05]	0.06

AVB, atrio-ventricular block; BMI, body mass index; CI, confidence interval; eGFR, estimated glomerular filtration rate; GCM, giant cell myocarditis; HR, hazard ratio; LV EF, left ventricle ejection fraction; NYHA, New York Heart Association; NT-proBNP, N-terminal pro-B-type; RV, right ventricular; VT, ventricular tachycardia

## Discussion

In this retrospective study of Swedish patients with CS and GCM, both entities presented with a similar spectrum of cardiovascular manifestations, but displayed different clinical courses and outcomes. Patients with GCM had a more rapid and fulminant course, higher levels of circulating biomarkers, more often biventricular systolic HF, and worse outcomes compared with CS patients. The association of GCM with inferior outcome remained significant even after adjustment for markers of cardiac dysfunction.

CS can share histopathological and clinical features with GCM, making correct diagnosis challenging [7, 22]. Conduction abnormalities, ventricular arrhythmias, and HF have been reported as initial clinical manifestations of both entities, albeit the presentation and course seem to be more aggressive in GCM [22, 23]. Non-necrotizing myocardial granulomas and fibrosis are considered as the histological hallmarks of CS while prominent necrosis together with multinucleated giant cells and eosinophils are the features of GCM [16, 23, 24].

How the clinical differences relate to variable host immunological factors or to different environmental exposures is still unclear [22]. There have been several reports of patients possibly having both conditions [25, 26]. Biopsy evidence of extracardiac sarcoidosis was reported in 5–10% of patients with a cardiac biopsy suggesting GCM [8, 27]. Roberts et al. reviewed 113 autopsy patients and concluded that the reported cases of GCM with granulomas outside the heart were more likely to be CS [27]. Based on these findings, it was proposed that GCM could be a subtype of sarcoidosis-like disease that only involves the heart [7, 8].

Whether CS and GCM are different disease entities or two expressions of a single disorder is still a matter of debate [8]. Our findings suggest that these are two separate clinical and pathologic entities, with different clinical courses, often needing different intensity of immunosuppression. In the present cohort, besides a more aggressive course, severe right ventricular dysfunction, diastolic LV dysfunction, and mild pulmonary hypertension were more common in GCM patients. Thus, not surprisingly, poorer outcomes were observed in patients with GCM compared to those with CS with respect to both need for HTx and death. Hence, GCM remained significantly associated with poor outcome even after adjustment for markers of severity of cardiac injury. In a study based on the Multicenter Idiopathic GCM Registry by Okura et al. cardiac histopathology and clinical features of 73 patients with GCM and 42 with CS were compared [16]. The 5-year transplant-free survival was 61% in CS versus only 10% in GCM, and, consistent with our findings, patients with GCM had a more fulminant disease course. Nordenswan et al. compared characteristics and outcomes of 311 CS patients and 25 GCM patients from the MIDFIN (Myocardial Inflammatory Diseases in Finland) Study [7]. Still, the severity of acute cardiac injury and dysfunction, as well as, the long-term outcome were worse in GCM with a 5-year estimate of event-free survival of 27% as compared with 77% in CS [7].

The description of echocardiography and CMR findings in patients with CS and GCM performed in the present study adds to the literature by showing that a biventricular dysfunction is more common in GCM than CS. A rapid deteriorating biventricular function shortly after disease onset, in combination with high levels of circulating natriuretic peptides and troponins, should raise the suspicion of GCM.

Although a female preponderance (around 60–80%) was usually reported in previous studies, [7, 28–30] a male predominance was revealed in 115 cases of GCM and CS from the United States and Japan (52 and 60%, respectively) [16]. In our cohort, a male predominance was found. Also, male gender was a strong independent predictor of worse outcome, though no significant difference was found with respect to age or comorbidities between men and women, apart from thyroid disease being more frequent among women (data not shown). Sex differences in outcome might be, in part, related to testosterone levels. It has been suggested that testosterone promotes myocarditis, including GCM, through the soluble suppressor of tumorigenicity-2 (sST2) pathway [31]. Increased sST2 levels in male mice correlate with poorer heart function, and male mice develop more severe myocarditis and progress to chronic HF more often than female mice [31]. Accordingly, elevated soluble ST2 was associated with an increased risk of HF in men ≤ 50 years old, with clinically suspected and biopsy-confirmed myocarditis [32]. There are thus plausible pathophysiological explanations for the negative relationship between male gender and outcome observed in our uni- and multivariable analysis.

During the 30-year study period, there was a steady increase of patients diagnosed with CS, while the number of patients diagnosed with GCM was stable. Findings by other groups suggest that the true prevalence of CS may be several times higher than the number of clinical diagnoses [27, 33]. We assume that a combination of improved diagnostic methods, heightened resolution in the pursuit of diagnosis and greater awareness of this disease, rather than a true increase in disease incidence, may explain these findings [14, 34]. On the other hand, a rather constant number of reported GCM cases could be due to the fulminant and inexorable progression of this disease, limiting the opportunity to perform a complete diagnostic work-up.

### Strengths and limitations

Several limitations should be mentioned. First, due to the retrospective nature of this study, caution must be applied before extrapolating results to other centers. Second, although most patients with clinically suspected are referred to our sarcoidosis unit from all the hospitals in the Västra Götaland region, a selection bias might be present. As our center is a tertiary hospital, many of the patients had more severe and advanced-stage disease. Third, we did not succeed to collect CMR and ^18^F-FDG PET data in all cases mainly due to the hemodynamic instability of some patients and the revision of diagnostic criteria during the study period not allowing for a standardized diagnostic work-up. In the light of the rarity and difficulty in diagnosing these disorders, the major strength of our work is the large study population and the detailed clinical data, with no missing cases at follow up. All GCM diagnoses were based on myocardial histology and the biopsies were re-analyzed by a highly-experienced cardiac pathologist. Similarly, 50% of the CS cases were diagnosed based on myocardial histology. Still, although the information is gathered from a single center, as the one of two-transplantation centers in Sweden, the patients referred to our center represent a large part of the country.

## Conclusion

Despite similar presenting manifestations, patients with GCM face a more aggressive clinical course as compared to those with CS and experience poorer outcomes. Severe biventricular failure along with presence of pulmonary hypertension and high levels of circulating natriuretic peptides should raise the suspicion of GCM. Whether CS and GCM could be different spectra of the same disease remains a matter of controversy.

## Supplementary Information

**Additional file 1**.** Table S1**. Clinical and demographic characteristics at presentation of the patients included in the survival analysis.

**Additional file 2**.** Table S2**. Findings from electrocardiography, echocardiography, and cardiovascular magnetic resonance imaging examinations at presentation of the patients included in the survival analysis.

## Data Availability

All data generated or analysed during this study are included in this published article and its supplementary information files.
