# Supplementary material for: Diagnosis, management, and outcome of cardiac sarcoidosis and giant cell myocarditis: a Swedish single center experience
Source: BMC Cardiovasc Disord. 2022 Apr 26;22:192. doi: 10.1186/s12872-022-02639-0 (PMC9044839; doi:10.1186/s12872-022-02639-0)
Supplement: Supplementary file 1 — Additional file 1. Table S1. Clinical and demographic characteristics at presentation of the patients included in the survival analysis. [file 12872_2022_2639_MOESM1_ESM.docx]

| **Additional file 1: Table S1**: Clinical and demographic characteristics at presentation of the patients included in the survival analysis. | | | | |
| --- | --- | --- | --- | --- |
|  | **All patients**  **(n=84)** | **CS**  **(n=65)** | **GCM**  **(n=19)** | ***p*** |
| Age (years) | 55 (48-60) | 56 (48-60) | 54 (46-67) | 0.106 |
| Female Gender | 28 (33) | 18 (28) | 9 (47) | 0.086 |
| BMI (kg/m^2^) | 26.3 (23.1-30.2) | 27.3 (24.4-30-5) | 22.7 (20.8-26.2) | 0.011 |
| NYHA class ≥ III | 31 (37) | 17 (26) | 14 (74) | <0.001 |
| **Comorbidities and laboratory findings** | | | | |
| Hypertension | 28 (33) | 24 (36) | 5 (26) | 0.299 |
| Diabetes mellitus | 5 (6) | 5 (8) | 0 | 0.272 |
| Previous CVD | 10 (13) | 10 (5) | 1 (5) | 0.238 |
| Thyroid disease | 11 (14) | 8 (12) | 3 (16) | 0.465 |
| Autoimmune Disease | 5 (6) | 0 | 4 (21) | 0.002 |
| NT-proBNP (pg/mL) | 958 (259-5752) | 698 (203-1450) | 8060 (2830-28500) | 0.003 |
| Troponin T (ng/L)^*^ | 31 (10-218) | 18 (7-55) | 473 (128-1270) | <0.001 |
| Creatinine (mg/dL) | 95 (80-119) | 91 (74-110) | 134 (100-178) | <0.001 |
| eGFR (ml/min/1.73m^2^) | 73 (55-81) | 75 (58-83) | 54 (33-77) | 0.013 |
| **Prevalent cardiac manifestations at presentation** | | | | |
| Heart failure | 26 (31) | 20 (30) | 6 (32) | 0.562 |
| Sustained VT or VF | 22 (26) | 14 (21) | 8 (42) | 0.066 |
| High-grade AVB | 17 (21) | 16 (24) | 1 (5) | 0.059 |
| Sudden cardiac arrest | 6 (7) | 5 (8) | 1 (5) | 0.596 |
| Chest pain | 7 (8) | 4 (6) | 3 (16) | 0.183 |
| Other symptoms or signs^¶^ | 6 (7) | 6 (9) | 0 | 0.208 |

Data are numbers (%) of cases; medians (Interquartile Range)

^#^including: supraventricular arrhythmias, transient ischemic attack, .^*^data reported on 46 patients (55% of the entire cohort, 48% of the CS group, 79% of the GCM group)

^¶^including: fever, fatigue, and dizziness; AVB, Atrio-ventricular block; BMI, Body mass index; CVD, Cardiovascular diseases; CS, Cardiac sarcoidosis; eGFR, Estimated Glomerular filtration rate by CKD-EPI equation; GCM, Giant cell myocarditis; NYHA, New York Heart Association; VT, Ventricular tachycardia; VF, Ventricular fibrillation
