# Supplementary material for: Diagnosis, management, and outcome of cardiac sarcoidosis and giant cell myocarditis: a Swedish single center experience
Source: BMC Cardiovasc Disord. 2022 Apr 26;22:192. doi: 10.1186/s12872-022-02639-0 (PMC9044839; doi:10.1186/s12872-022-02639-0)
Supplement: Supplementary file 2 — Additional file 2. Table S2. Findings from electrocardiography, echocardiography, and cardiovascular magnetic resonance imaging examinations at presentation of the patients included in the survival analysis. [file 12872_2022_2639_MOESM2_ESM.docx]

| **Additional file 2: Table S2**  Findings from electrocardiography, echocardiography, and cardiovascular magnetic resonance imaging examinations at presentation of the patients included in the survival analysis | | | | |
| --- | --- | --- | --- | --- |
|  | **All patients**  **(n=84)** | **CS**  **(n=65)** | **GCM**  **(n=19)** | ***p*** |
| **ECG** |  |  |  |  |
| Atrial fibrillation | 1 (1) | 1 (1) | 0 | 0.396 |
| HR (bpm) | 63 (54-79) | 62 (50-75) | 75 (63-102) | 0.006 |
| PQ (ms) | 182 (163-216) | 188 (155-212) | 177 (163-224) | 0.761 |
| High-grade AVB | 17 (21) | 16 (24) | 1 (5) | 0.059 |
| Right BBB | 2 (23) | 16 (7) | 4 (27) | 0.616 |
| Left BBB | 11 (13) | 11 (18) | 0 | 0.073 |
| Q-wave | 5 (6) | 3 (5) | 2 (13) | 0.254 |
| Frequent PVCs | 16 (19) | 11 (18) | 5 (33) | 0.170 |
| **Echocardiography** | | | | |
| LV EF (%) | 45 (30-55) | 47 (36-55) | 30 (20-46) | 0.003 |
| LV ED diameter (mm) | 56 (50–62) | 56 (50–62) | 54 (50-61) | 0.596 |
| LV ED volume (ml) | 135 (100–178) | 155 (100–204) | 123 (99-147) | 0.063 |
| LV DD | 32 (38) | 19 (34) | 13 (72) | 0.005 |
| RV dysfunction | 24 (28) | 14 (25) | 10 (59) | 0.012 |
| sPAP (mmHg) | 30 (24-40) | 28 (22-38) | 40 (30-45) | 0.067 |
| **MRI** | | | | |
|  | N=53 | N=41 | N=12 |  |
| LV ED volume (ml) | 171 (134-234) | 162 (129-234) | 193 (159-249) | 0.101 |
| LV ES volume (ml) | 140 (85-183) | 88 (73-209) | 150 (105-157) | 0.450 |
| LV EF (%) | 44 (32-57) | 50 (36-61) | 30 (22-42) | 0.004 |
| RV ED volume (ml) | 162 (123-228) | 154 (96-218) | 188 (139-247) | 0.679 |
| RV ES volume (ml) | 108 (74-202) | 102 (57-229) | 119 (92-186) | 0.428 |
| RV EF (%) | 48 (26-60) | 52 (37-62) | 29 (24-45) | 0.025 |
| Delayed enhancement | 51 (96) | 40 (97) | 11 (92) | 0.623 |

Data are numbers (%) of cases; medians (Interquartile Range)

AVB, Atrio-ventricular block; BBB, Bundle branch block; HR, Heart rate; LV DD, Left ventricular diastolic

dysfunction; LV ED, Left ventricular end-diastolic; LV EF, Left ventricular ejection fraction; LV ES, Left

ventricular end-systolic; PVCs, Premature ventricular complexes; RV, Right ventricular; RV ED, Right

ventricular end-diastolic; RV EF, Right ventricular ejection fraction; RV ES, Right ventricular end-systolic
